# An Incident in Dr. Garretson’s Life

**Published:** 1897-07

**Authors:** G. Alden Mills

**Affiliations:** New York


### Domestic Correspondence.

### AN INCIDENT IN DR. GARRETSON'S LIFE.

NEW YORK, May 14, 1897.

TO THE EDITOR:

SIR,—The tribute paid to Professor Garretson in your May number, by Professor Guilford and others, called to mind a very pleasant incident in this connection that much endeared me to Professor Garretson. It came as a surprise to me.

In 1882 I had performed a very peculiar surgical operation before the New Jersey Society at Long Branch and an account of it appeared in the *Dental Cosmos*, contributed by the reporter of the Society, and greatly to my pleasure I received a congratulatory note from Professor Garretson. It was a kind and pleasant thing to do from a truly great man. He subsequently sent me an invitation to witness an operation by himself of removing a portion of the extreme end of the vertebral column with the surgical engine. There I met Drs. Agnew, Pancoast, and others of note, and lunched with them, feeling honored for such an opportunity.

This is my small tribute to his blessed memory.

G. Alden Mills.

